# Efficacy of Carboxytherapy Mask in Post‐Fractional Ablative Laser Recovery: A Randomized Pilot Trial

**DOI:** 10.1111/jocd.70481

**Published:** 2025-10-09

**Authors:** Steven Dayan, Catherine Carvajal, Nimit Gandhi, Lander McGinn, Mia Cirrincione, Jose Sanchez‐Perez

**Affiliations:** ^1^ DeNova Research Chicago Illinois USA

**Keywords:** carboxytherapy, fractional ablative laser resurfacing, laser resurfacing

## Abstract

**Background:**

Fractional ablative CO_2_ lasers are widely used for skin rejuvenation but are associated with post‐procedural erythema, crusting, tenderness, and downtime. Carboxytherapy masks, a modality utilizing carbon dioxide, have been suggested to enhance healing and aesthetic outcomes post CO_2_ laser treatment; however, controlled clinical data remain limited.

**Objective:**

To evaluate the efficacy and safety of a topical carboxytherapy mask in enhancing recovery, reducing adverse effects, and improving patient satisfaction following fractional ablative CO_2_ laser treatment.

**Methods:**

Ten subjects (aged 45–70 years, Fitzpatrick skin types I–III) undergoing full‐face fractional CO_2_ laser resurfacing were enrolled in this randomized pilot study. Participants were assigned to either the active arm (*n* = 8, carboxytherapy mask was applied pre‐ and post‐procedure and at designated intervals for 14 days) or placebo arm (*n* = 2, bland moisturizer). Outcomes included blinded investigator assessments of erythema, edema, crusting, healing rate, pigmentation, and wrinkle severity at Days 28 and 84; patient‐reported diaries of discomfort and healing parameters; and self‐assessments of satisfaction and global aesthetic improvement.

**Results:**

The active group demonstrated reduced erythema during the first week (mean scores: Day 1, 3.1 vs. 3.5; Day 7, 0.6 vs. 1.0 for placebo). By Day 7, healing surface area averaged 97% in the active group with no crusting. At Day 28, the active group exhibited fewer fine and coarse lines (mean coarse line score 2 vs. 4 in placebo). At Day 84, coarse line reduction and abnormal pigmentation improvement favored the active group (0.75 vs. 1.5, respectively). Patient‐reported tenderness, burning, and crusting were consistently lower in the active group, with higher satisfaction and confidence scores at both Day 28 and 84. No adverse effects were reported.

**Conclusion:**

In this pilot study, the carboxytherapy mask enhanced post‐laser healing, reduced erythema and discomfort, and improved long‐term wrinkle and pigmentation outcomes compared to placebo. These findings support the adjunctive use of the carboxytherapy mask to improve recovery and patient satisfaction after ablative CO_2_ laser resurfacing. Larger, controlled trials are warranted to confirm these results.

## 1 Background

Energy‐based skin rejuvenation technologies, like carbon dioxide (CO_2_) resurfacing lasers, achieve an improved appearance by causing a measured amount of thermal damage to the epidermis and heating the dermis, thereby inducing wound healing with a resultant brighter and more even skin texture. These ablative lasers, while effective, are not without significant side effects including redness, swelling, crusting, pain, and potentially skin discoloration. Given their efficacy, however, patients increasingly seek these minimally invasive treatments, underscoring the importance of appropriate post‐treatment care to minimize potential side effects.

Carboxytherapy, a therapeutic modality utilizing CO_2_ introduced into the skin through topical application or sterile microinjections, has garnered substantial attention within medical literature due to its diverse clinical applications. Initially explored for its potential in enhancing cutaneous microcirculation and tissue oxygenation [1], carboxytherapy has since expanded its scope to encompass a range of therapeutic indications, including chronic wound healing [2], lymphedema management [3], and dermatological interventions [4]. Studies suggest it possesses a wealth of benefits including antioxidant, vasodilatory, anti‐inflammatory, analgesic, promotion of neoangiogenesis, and regenerative processes [5, 6] to support the microvascular‐tissular unit and overall tissue health [1, 2, 3, 4]. Specifically, in the cosmeceutical context, though, this technique induces vasodilation and intradermal collagen reorganization, resulting in improved skin laxity and hyperpigmentation among several other indicators of skin aging [7]. Moreover, carboxytherapy masks have already been demonstrated to be efficacious for improvement of signs of healing after energy‐based skin rejuvenation procedures [8].

A topical carboxytherapy gel mask (CO_2_ Lift, Lumisque Inc., Weston, FL) has been developed and is utilized by several aesthetic practices as an over‐the‐counter cosmetic face mask to aid in skin recovery post‐procedure. While carboxytherapy masks have gained traction in clinical practice, the precise mechanisms underlying their therapeutic effects, as enumerated above, remain to be fully elucidated. Furthermore, variations in treatment protocols and dosing regimens highlight the need for standardized guidelines to optimize patient outcomes and ensure safety [5]. In light of these considerations, this study aims to investigate the impact of a carboxytherapy mask using a readily available gel mask on healing outcomes after fractional ablative laser skin rejuvenation, shedding light on its therapeutic potential and informing clinical practice.

## 2 Methods

### 2.1 Subjects

This study recruited and consented 10 healthy female subjects aged 45–70 years, with Fitzpatrick skin types I–III, with mild to moderate photoaging, who were already intended to undergo full face CO_2_ ablative laser treatment. Subjects were contacted via telephone and invited to enroll. Participants were excluded from the study if they met any of the following criteria; 1. Any dermatological disorder, which in the investigator's opinion, may interfere with the accurate evaluation of the subject's skin characteristics, except for the conditions associated with sensitive skin or photoaging skin, 2. Subjects who are pregnant or lactating, 3. Subjects with history of hypertrophic scarring or keloids, 4. Subjects who do not agree to refrain from direct sun exposure during the study duration, 5. Subjects who are unwilling or unable to comply with the requirements of the protocol, 6. Subjects with any planned surgeries and/or invasive medical procedures during the study, 7. Subjects currently receiving any anticancer, immunosuppressive treatments/medications (e.g., azathioprine, belimumab, cyclophosphamide, etanercept, adalimumab, mycophenolate mofetil, methotrexate, prednisone, infliximab, and ustekinumab), or radiation. The participants were randomized into either the active group with application of a carboxytherapy mask, or to the placebo group with application of solely Vanicream Daily Facial Moisturizer. Two of the ten subjects were randomly assigned to the placebo group via a computerized random number generator to minimize allocation bias.

### 2.2 Carboxytherapy Mask

The carboxytherapy mask was prepared by mixing the contents of two packages containing a gel and activator into a small bowl for 30 s–1 m. The treatment area was then covered with a thick layer of gel, which was left on there for an hour until it solidified and was removed.

### 2.3 Fractionated CO_2_ Laser

All CO_2_ laser treatments were administered by a licensed RN in accordance with the study protocol and under the oversight of a physician. Energy: 100 mJ; Rate: 100 Hz; CPG Pattern: 3/5/1; Repeat Delay: 0.5 s.

### 2.4 Study Design

Subjects washed their faces, and topical anesthesia (23% lidocaine/7% tetracaine ointment) was applied. The placebo group only received topical anesthesia 1 h prior to the administration of laser treatment. For the active group, the carboxytherapy mask was immediately applied on top of the topical anesthesia cream for 1 h prior and again immediately post‐procedurally. The active group reapplied the carboxytherapy mask at 24‐, 48‐, and 72‐h marks and then on Days 7 and 14 post‐procedurally, for a total of seven treatments. In conjunction with the carboxytherapy mask, the active group used Vanicream Gentle Facial moisturizer as needed on Days 0–14. Meanwhile, the placebo group applied only the Vanicream Gentle Facial moisturizer twice daily for Days 0–14. Starting Day 2, both groups cleansed with Vanicream Gentle Facial Cleanser prior to applying their respective study products. Both groups applied an identical SPF30 facial sunscreen daily on Days 7–28.

Subjects were evaluated at clinic follow‐up visits on Days 1, 3, 4, 7, 28, and finally 84. At all visits, 2D photographs were taken and questionnaires were completed. The questionnaires assessed a participant's self‐perception of healing parameters, wrinkle severity, global aesthetic improvement score (GAIS), and satisfaction. Investigator healing assessments were performed live at each visit. A percentage of surface area healed and an overall healing grade on a 5‐point scale comparing the current healing experience to the investigator's past was obtained. Investigator‐assessed healing parameters included erythema, edema, crusting, and exudation on a 5‐point scale. Additionally, at Day 28 and Day 84, photodamage/wrinkle, fine lines, coarse lines, abnormal pigmentation, and a global assessment on a 10‐point scale were performed by the investigator. Subjective healing parameters included erythema, swelling, crusting/flaking, bruising, itching, tenderness, and burning/stinging experienced on a standard 10‐point scale daily via diary entries measured at Days 1–14. At Days 28 and 84, a self‐assessment score of wrinkle severity and satisfaction was also collected, followed by global aesthetic improvement score (GAIS). Subjects were asked the degree to which they agreed with the following statements on a 5‐point scale: “Improved the evenness of my skin tone,” “Made my skin look brighter and more healthy,” “Made my skin look more youthful,” “Made me feel more confident in the way my skin looks,” “I would continue using this regimen,” “I would recommend this treatment to other.” All data were analyzed using a Wilcoxon signed rank test and a sign test. The noninvasive parametric data were analyzed using a paired *t*‐test.

## 3 Results

### 3.1 Investigator Assessments

**FIGURE 1:**
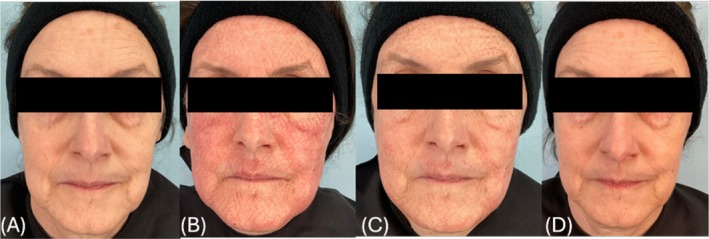
Subject in the active group (A) preprocedure and at (B) postprocedure Day 1, (C) postprocedure Day 3 and (D) postprocedure Day 7.

**FIGURE 2:**
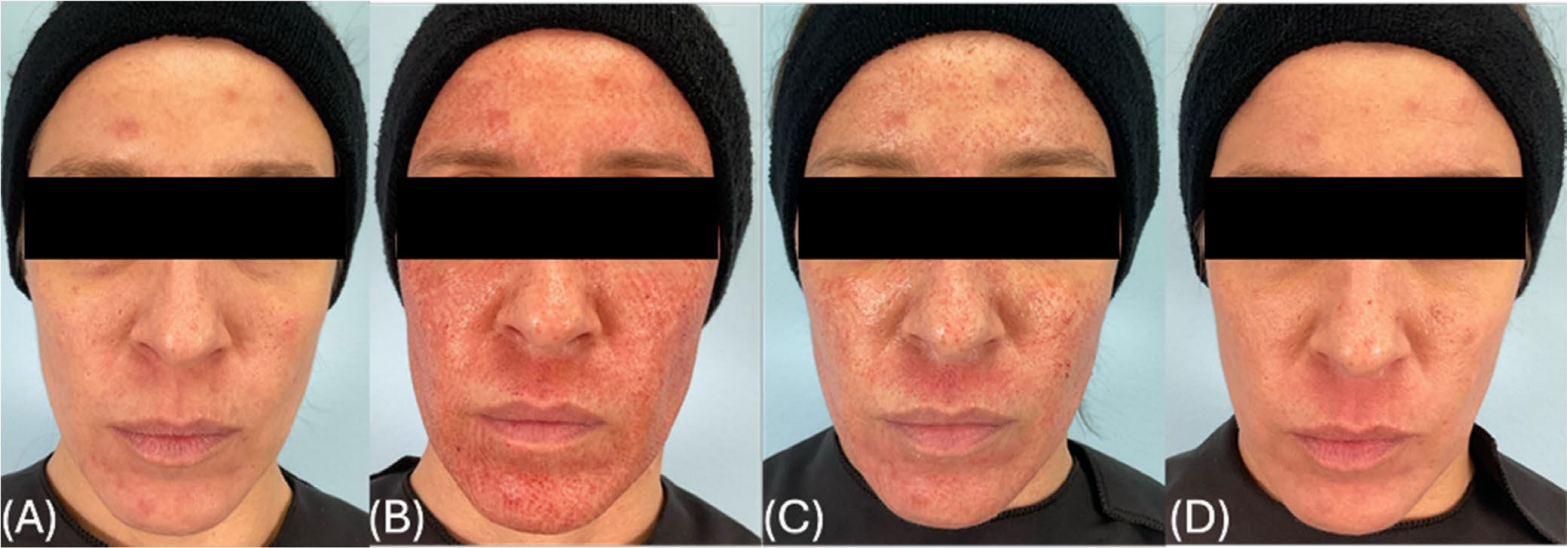
Subject in the placebo group (A) preprocedure and at (B) postprocedure Day 1, (C) postprocedure Day 3, and (D) postprocedure Day 7.

Data from the 10 enrolled subjects was analyzed. A blinded investigator rated the active group as having less erythema during the first post‐procedure week (Figure 1). On post‐procedure Day 1, the active group scored an average of 3.1 out of 5, whereas the placebo group (Figure 2) scored an average of 3.5. On Day 3, the active group received an average erythema score of 2.25, while the placebo group received a score of 2.5. Impressively, by Day 7, the amount of residual erythema in the active group was nearly half as much as in the placebo group with scores of 0.6 versus 1, respectively. The active group also showed improved overall healing assessments compared to the placebo group, with a healed surface percent score of 97% and no evident crusting at Day 7. On Day 28, the active group on average showed fewer fine lines and half as many coarse lines with a score of 2 on a 0–9 scale as opposed to the placebo (score of 4). At Day 84, a significant difference for coarse lines was gathered favoring the active group. Globally, the active group also had less photodamage, as assessed by raters on Day 28. Improved facial aesthetics continued for the active group long‐term. The active group showed a lower abnormal pigmentation average score of 0.75 at Day 84 (final follow‐up), which was almost half of what the placebo group showed, which obtained an average score of 1.5. Additionally, coarse line averages remained consistently better for the active group but worsened for the placebo group which had an average score of 5.5 at Day 84.

### 3.2 Subject Take Home Diary

**FIGURE 3:**
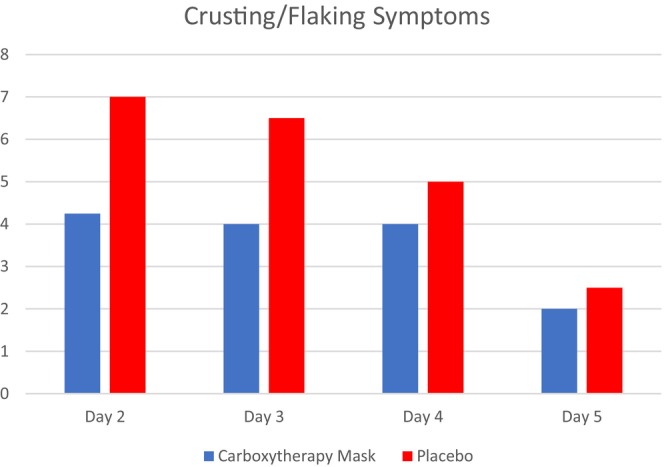
Subjects in active and placebo groups reported crusting and flaking symptoms, documented through take‐home diaries, observed one week following ablative CO_2_ laser treatment.

The active group felt less tenderness, crusting/flaking, itching, and burning/stinging the first week post laser procedure compared to the placebo group (Figure 3). On post procedure days 1 and 2, study participants rated tenderness as a 5.3 and 3.2 on average, respectively. By comparison, the placebo group rated tenderness as an 8.5 and a 6.5 on average for days 1 and 2. By recording an average score of 0.8 versus 1.5 on Day 7, the active group showed almost half the level of tenderness compared to the placebo group. Crusting/flaking and itching scores remained consistently lower for the active group on days 2–5 post procedure (Figure 3). On Day 1, subjects in both groups reported the highest levels of burning and stinging; however, the active group's score was much lower, at 6.1, compared to 8.5 for the placebo group.

### 3.3 Subject Satisfaction

**FIGURE 4:**
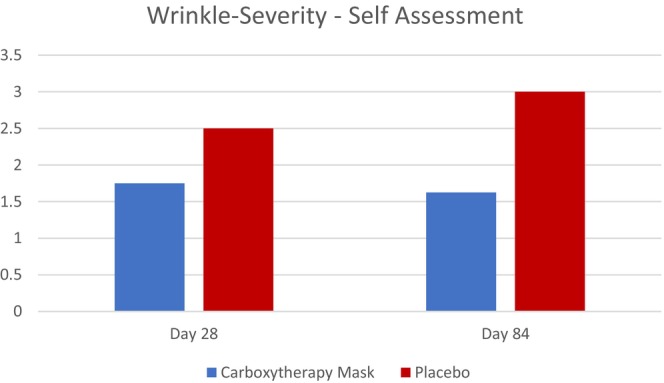
Active and placebo subjects reported Wrinkle Severity score at Day 28 and Day 84 following CO_2_ laser treatment.

**FIGURE 5:**
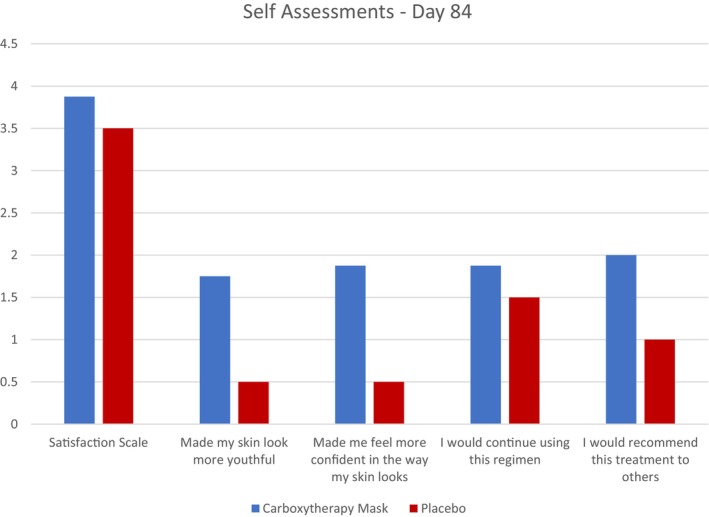
Active and placebo subjects' reported product and skin satisfaction, confidence, and likelihood of continued product use at Day 84 post ablative CO_2_ laser treatment.

The active group reported higher rates in their appearance, results, and confidence over the placebo group at Days 28 and 84. On Day 28, the active group reported a lower wrinkle severity score compared to placebo (1.75 vs. 2.5) (Figure 4). On average, the active group also reported higher overall satisfaction levels, with patients feeling more confident about the way their skin looked. At Day 84, differences in the active versus placebo group were even more pronounced. The active group reported they would be more likely to continue using the carboxytherapy mask and that they would recommend it more to others over standard of care. The active group also felt their skin appeared more youthful and with an even greater reduction in their wrinkle severity compared to Day 28 (Figure 5). By contrast, the placebo group reported an increase in wrinkle severity at Day 84 from Day 28 (Figure 4), consistent with coarse line investigator assessment scores. Subjects reported no difference between the active and placebo groups for skin tone evenness and brightness at Day 84.

## 4 Discussion

Patients pursue ablative lasers for improvements in the global appearance of their skin. These lasers, however, warrant a certain period of downtime for the best healing and recovery. The carboxytherapy mask findings in this study suggest that this downtime period and patient symptomatology are lessened during the first postprocedural week. With speedy healing and improved self‐assessments, a higher patient satisfaction outcome was observed in the active group over subjects that applied the standard of care moisturizer. The advantages of the carboxytherapy mask extended beyond general contentment. There was also a psychological benefit, where users felt more confident about the way their skin looked and were likely to recommend the carboxytherapy mask to others (Figure 5).

Carboxytherapy masks are suggested to have their beneficial healing properties due to a series of events that increase circulation and peripheral blood flow. When CO_2_ reacts with water, carbonic acid is created, which leads to a lower skin pH [9]. The decrease in pH lessens the hemoglobin affinity toward oxygen, resulting in the release of oxygen into the tissues [9]. The carboxytherapy mask can bypass the injury phase of wound healing and move directly into the repair phase by locally stimulating growth factors and, primarily, reducing the inflammatory response at the wound site. This results in a quicker recovery, lessening erythema, stimulating neo‐collagenesis, repairing damaged areas, and preventing post‐inflammatory pigmentation [8].

In an effort to increase treatment satisfaction, prior research has been done investigating fears patients may have before undergoing an ablative laser treatment. Among the most cited was post‐procedural redness and pain [10]. This study identified that post‐procedural erythema was diminished in the active group (Figures 1, 2, 3), and these same patients reported less tenderness compared to their placebo counterparts. These results suggest that using a carboxytherapy mask could help lessen these fears and improve patient satisfaction. Additionally, severe wrinkle appearance progressively improved, as seen on Day 28 and Day 84 (Figure 4), indicating that the carboxytherapy mask may be an additional component to augment the effectiveness of laser treatments to achieve noticeable results and increase patient satisfaction.

It should be noted that while the results of this study are promising, the number of patients is ultimately a limited data set. The placebo group of two individuals was assessed at numerous intervals, as were the active, to obtain numerous data points. The results from this pilot study provide a launch pad for future research into this product or others like it to better understand how carboxytherapy masks can augment well‐established means of aesthetic improvement, such as laser resurfacing. The results of this study are not representative of the whole population of those undergoing ablative lasers, as not all Fitzpatrick skin types were represented. It does, however, stand to reason that this product poses minimal to no risk for worsening of healing following these treatments, and may in fact speed healing and improve satisfaction with the procedure, the healing period, and the aesthetic outcome. Further research will need to be conducted in topical treatments of carboxytherapy to firmly establish such a link.

## 5 Conclusion

This pilot study investigated the use of a topically applied gel mask modality for administering carboxytherapy immediately preceding, and in the recovery period following fractional ablative CO_2_ laser. The results indicate that the product investigated, a carboxytherapy mask, is safe compared to a placebo product, a readily available bland moisturizer. Participants within the active group unanimously indicated a high level of satisfaction with their results. Moreover, the product in question yielded impressive results for postprocedural wound healing, with improved speed of resolution of erythema and crusting, and improved end‐point reduction in coarse lines and abnormal pigmentation. Offering patients a readily available topical carboxytherapy mask offers cosmetic experts a means to reduce some of the most common complications of laser treatments.

## Author Contributions

All authors contributed to the conception, design, execution of the trial, data collection, analysis, and drafting of the manuscript. All authors reviewed and approved the final version of the manuscript.

## Ethics Statement

The study was conducted in accordance with the principles of the Declaration of Helsinki and approved by Advarra Institutional Review Board on February 23, 2024 (approval number: Pro00077635).

## Consent

Written informed consent and photography consent were obtained from all subjects prior to participation in the study.

## Conflicts of Interest

The authors declare no conflicts of interest.

## Data Availability

The data supporting the findings of this study are available from the corresponding author upon reasonable request.
